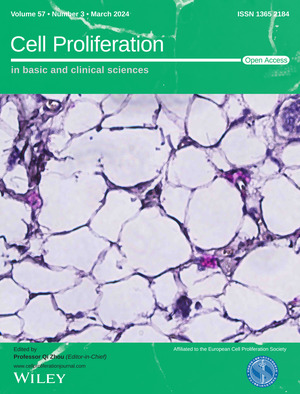# Additional Cover

**DOI:** 10.1111/cpr.13629

**Published:** 2024-03-01

**Authors:** Nahyun Choi, Juyeong Hwang, Doo Yeong Kim, Jino Kim, Seung Yong Song, Jong‐Hyuk Sung

## Abstract

The cover image is based on the Original Article *Involvement of DKK1 secreted from adipose‐derived stem cells in alopecia areata* by Nahyun Choi et al., https://doi.org/10.1111/cpr.13562.